# Aging-Induced Proteostatic Changes in the Rat Hippocampus Identify ARP3, NEB2 and BRAG2 as a Molecular Circuitry for Cognitive Impairment

**DOI:** 10.1371/journal.pone.0075112

**Published:** 2013-09-19

**Authors:** Philipp Ottis, Bianca Topic, Maarten Loos, Ka Wan Li, Angelica de Souza, Daniela Schulz, August B. Smit, Joseph P. Huston, Carsten Korth

**Affiliations:** 1 Department of Neuropathology, Heinrich Heine University of Düsseldorf, Düsseldorf, Germany; 2 Center for Behavioral Neuroscience, Department Experimental Psychology, Heinrich Heine University of Düsseldorf, Düsseldorf, Germany; 3 Department of Molecular and Cellular Neurobiology, Faculty of Earth and Life Sciences, Center for Neurogenomics and Cognitive Research, Neuroscience Campus Amsterdam, VU University, Amsterdam, The Netherlands; 4 Synaptologics B.V., Amsterdam, The Netherlands; Universidad de Sevilla, Spain

## Abstract

Disturbed proteostasis as a particular phenotype of the aging organism has been advanced in *C. elegans* experiments and is also conceived to underlie neurodegenerative diseases in humans. Here, we investigated whether particular changes in non-disease related proteostasis can be identified in the aged mammalian brain, and whether a particular signature of aberrant proteostasis is related to behavioral performance of learning and memory. Young (adult, n = 30) and aged (2 years, n = 50) Wistar rats were tested in the Morris Water Maze (MWM) to distinguish superior and inferior performers. For both young and old rats, the best and worst performers in the MWM were selected and the insoluble proteome, termed aggregome, was purified from the hippocampus as evidence for aberrant proteostasis. Quantitative proteomics (iTRAQ) was performed. The aged inferior performers were considered as a model for spontaneous, age-associated cognitive impairment. Whereas variability of the insoluble proteome increased with age, absolute changes in the levels of insoluble proteins were small compared to the findings in the whole *C. elegans* insoluble proteome. However, we identified proteins with aberrant proteostasis in aging. For the cognitively impaired rats, we identified a changed molecular circuitry of proteins selectively involved in F-actin remodeling, synapse building and long-term depression: actin related protein 3 (ARP3), neurabin II (NEB2) and IQ motif and SEC7 domain-containing protein 1 (BRAG2). We demonstrate that aberrant proteostasis is a specific phenotype of brain aging in mammals. We identify a distinct molecular circuitry where changes in proteostasis are characteristic for poor learning and memory performance in the wild type, aged rat. Our findings 1. establish the search for aberrant proteostasis as a successful strategy to identify neuronal dysfunction in deficient cognitive behavior, 2. reveal a previously unknown functional network of proteins (ARP3, NEB2, BRAG2) involved in age-associated cognitive dysfunction.

## Introduction

The unraveling of the specific 'pathophysiology' of natural, non disease-associated brain aging is only emerging. Whereas general principles of cellular aging like telomere shortening [1], mitochondrial dysfunction leading to increased intracellular oxidative stress [2], or the involvement of insulin/IGF-1 (insulin-like growth factor 1)-like signaling [3] are well established, the specific molecular features of cellular aging in post-mitotic neurons of the brain are still not well understood.

Changes in protein homeostasis (proteostasis), i.e. the orderly life cycle of synthesis and degradation of proteins, have been described for the aged mammalian brain in terms of gene expression [4], epigenetic changes [5], and protein composition (reviewed by VanGuilder and Freeman in 2011 [6]). Proteomic changes comparing aged and young rodents primarily have been assigned to cellular processes such as glucose metabolism [7,8,9,10,11,12], signal transduction [7,8,9,10,11,13], oxidative stress [9,13], and cytostructure regulation [8,12]. Changes in the expression of proteins that are involved in synaptic processes appear to be more specific to changes in cognition rather than aging [14,15,16,17]. Peter Douglas and Andrew Dillin reviewed the potential effects of age-associated proteostasis changes on neuronal health [18].

Studies on the nematode *Caenorhabditis elegans* [19,20] revealed increases in the overall content of insoluble proteins with age in the whole organism. Subsequent experiments demonstrated that RNAi knockdown of some of the identified insoluble proteins increased the worms’ lifespan [19]. This suggested that a decreased clearance of insoluble proteins may contribute to age-associated pathophysiology.

These findings also indicated that, at least in *C. elegans*, the mechanisms for quality control in proteostasis undergo an age-associated decline independent of any disease. Consequently, the analysis of specific proteins accumulating as a result of clearance dysfunction may reveal insights into the cellular mechanisms of neuronal aging, and provide potential targets for therapeutic intervention. In humans, one phenotype associated with brain aging is mild cognitive impairment (MCI) [21,22]. MCI is defined as a decrease in cognitive abilities in elderly subjects that is clearly discernible but not yet interfering with tasks of daily life [22]. As such, this condition often precedes Alzheimer’s disease [23,24].

As humans and animals age, individual differences become apparent across various behavioral domains [25,26]. While some aged subjects maintain performance levels comparable to that of young ones, termed successful or healthy aging [27,28], a fraction of aged individuals show impaired performances. This spontaneously arising increase in variability, found in aged cohorts of outbred rat strains, has been extensively studied in relation to learning and memory in aged rodents (e.g. [29,30,31,32]).

Here, the Morris water maze (MWM) represents a widely used task to assess individual differences in aging-related decrements in spatial learning and memory, with the spatial performance in this task being dependent on the functional integrity of the hippocampus [33].

In this study we aimed to answer 1. whether aging-associated disturbances in proteostasis, reflected by the segregation of distinct proteins into the insoluble proteome, can be observed in the rat brain, and 2. whether such segregation of certain insoluble proteins is functionally related to changes in learning and memory in aged rats.

Using quantitative proteomics, we demonstrate an age-dependent change in hippocampal proteostasis, indicated by compositional changes in the insoluble proteome, termed aggregome. Focussing on changes specific for the aged rats showing impaired performance compared to their age-matched superior performers, we identify a molecular circuitry related to synaptic plasticity.

## Materials and Methods

### Animals

The present study continues a report of results presented in Schulz et al. (2007) [34] using a subset of the same animals. Drug-naive adult (n = 30; 3 months) and aged (n = 50, 24 months) male outbred Wistar rats were obtained from the animal facilities of the University of Düsseldorf and were maintained under a reversed light-dark cycle (lights went on at 7: 00 pm for 12 h). They were housed in standard Macrolon cages of Type IV in groups of 2-3 old or 5 young animals per cage, and had free access to water and standard laboratory chow (Ssniff Spezialdiät). Over a period of three months the animals were behaviorally characterized by assessing their performance in the open field test, black-white box, elevated plus maze as well as learning and extinction trials in the Morris water maze (MWM). Behavioral testing was conducted during the dark period between 09:00 am and 06:00 pm and took place every 48 h (see also Schulz et al., 2007 [34]). All experiments were carried out in accordance with and approved by the German Animal Protection Law (Bezirksregierung Düsseldorf), as well as National and European Regulations.

### Morris water maze (MWM)

The procedure, experimental design and water maze apparatus have been described in detail elsewhere [34]. Briefly, the water maze consisted of a black circular swimming pool made of polyethylene that was filled with water (20 ± 1 °C) up to a depth of 30 cm. The diameter of the pool was 185 cm. For the cued version of the water maze a 0.5 cm diameter metal peg (height: 22 cm) with black and white stripes was fixed onto the circular platform (18 cm in diameter, 1.5 cm under the water surface level) with a clip and tagged with vanilla aroma. Within one day, rats were released into the water maze for four successive trials with the platform cued. If a rat did not escape onto the platform within 1.5 min, it was gently guided to it by the experimenter. Two days later, the animals were trained in the hidden platform place version of the water maze for 6 days with two training trials per day (one in the morning and one in the afternoon). During this phase, the platform was fixed 1.5 cm below the water surface in the center of one quadrant of four equally large virtual quadrants of the maze. The platform location was randomly varied between all rats, but was maintained in a fixed location for a given rat during each task. A trial ended either when a rat escaped onto the hidden platform, or after 2.5 min had elapsed. After each training trial in the water maze, the animals were dried under a red-light heating lamp, before being returned to their home cages.

The behavioral analysis during the acquisition trials for each rat comprised the distance to platform (cm) and the time spent within the platform quadrant (PQ, expressed as percentage of total trial duration) as well as the swimming speed (cm/sec), which were automatically recorded via the EthoVision tracking software (Version 3, Noldus, Wageningen, The Netherlands).

Since 11 animals (10 aged and 1 adult) exhibited obvious signs of physical weakness (such as body tumors, eye infection) during the course of experimental testing, their data were excluded from the behavioral analyses, resulting in aged: *n* = 40 and adults: *n* = 29.

### Clustering of animals according to their learning abilities

In the present report, we examined whether subgroups of superior and inferior learners in the water maze also exhibited differences in the insoluble hippocampal proteome. For classification into superior *vs.* inferior learners, we calculated the mean distance to the platform over all hidden platform place-learning trials for each rat to establish an overall score of learning performance. In resemblance to other studies on individual differences in learning and memory [30,35,36] the animals were ranked according to their overall score. This was done separately for each age group. Of each age group, 8 of the best performers were assigned to the "superior" group and 8 of the worst performers to the "inferior" group. However, the 15% of animals on each extreme side of the median were excluded in order to omit animals suggestive of any possibly unidentified disease or motoric disabilities, and also in order to reduce sampling bias.

### Statistical analysis of behavioral data

Data are presented as mean ± standard error of the mean (SEM). For the water maze, the mean curve level A_0_, assessed as total mean distance to platform, as well as the time spent in the platform quadrant (expressed as percentage of trial duration) was taken as an index of the average performance, and the linear trend component a_1_, describing the slope of the curve, was calculated as an estimate of the rate of behavioral change over the course of training [37]. Three-way repeated measures analyses of variance (ANOVAs) with pairwise multiple comparisons using Bonferroni adjustment were conducted for statistical analysis, with ‘age’ and ‘learning performance’ (superior and inferior learners) as between-groups factors and days as the repeated measures factor for the water maze acquisition (computed on mean of trials per day) data. For the cued version of the water maze, trials were used as the repeated measures factor. When appropriate, t-tests for independent groups were carried out using the A_0_ and a_1_ values to determine differences between superior and inferior learners within each age group and also superior and inferior learners between the age groups. The level of significance was set to p ≤ 0.05.

### Tissue and protein extraction

From a total of 32 animals comprising four groups (8 ‘adult inferior’, 8 ‘adult superior’, 8 ‘aged inferior’, and 8 ‘aged superior’) hippocampi were dissected, homogenized and aliquoted. Each rat hippocampus was processed individually in a blinded approach and in random order. One aliquot each, representing 20 mg of tissue, subjected to purification of detergent-insoluble proteins according to a protocol modified from Leliveld et al., 2008 [38] and Ottis et al., 2011 [39]. Briefly, the homogenates, supplemented with 2 mM phenylmethylsulfonyl fluoride (PMSF) and 1 x cOmplete, EDTA-free Protease Inhibitor Cocktail (Roche Applied Science, Mannheim, Germany), were incubated overnight at 4 °C in the presence of DNase I to degrade all DNA present in the sample. The next day, the aggregome was purified via three subsequent ultracentrifugation steps, each at 100.000 x g. Two subsequent centrifugation runs were carried out in a high-density sucrose buffer (1.1 M and 1.6 M), followed by one step in high salt buffer (1.5 M NaCl). Apart from the high salt treatment, all steps were carried out in the presence of 1.0% nonidet-P40 (NP-40) and 0.2% N-lauroylsarkosine (sarkosyl). Subsequently, the resulting pellet was washed twice in HEPES-buffer (50 mM 4-(2-hydroxyethyl)-1-piperazineethanesulfonic acid, pH 7.5) to remove salts and detergents incompatible with iTRAQ-experiments. All centrifugation steps were carried out using a TLA-55 rotor and 1.5 mL ultracentrifugation tubes (Beckman Coulter, Krefeld, Germany).

### Labeling for quantitative mass spectrometry

The pellets were dried in a speedvac and proteins were then denatured in 30 µL of 0.5 M triethyl ammonium bicarbonate (TEAB), 6 M urea, 0.8% RapiGest SF Surfactant (Waters Corporation, Milford, MA) and subjected to vigorous shaking at 25 °C for 10 min. After addition of 2 µL reducing reagent (supplied with the iTRAQ Reagent-Multiplex Buffer Kit; AB SCIEX, Darmstadt, Germany), samples were incubated at 27 °C for 2 hours with alternating shaking and pause intervals of 10 sec and 1 min, respectively. Subsequently, the reduced, free cysteines were blocked by addition of 1 µL of cysteine-blocking reagent (AB SCIEX, Darmstadt, Germany) and incubation with shaking at 25 °C for 10 min. For tryptic digestion, hydrolyzed, sequencing grade modified trypsin (Promega, Mannheim, Germany) was re-solubilized in 0.5 M TEAB to 0.4 mg/mL and 10 µL were added to each protein sample, followed by incubation at 37 °C overnight. Next day, 80 µL of HPLC-grade 2-propanol were added to each of the digests and tubes were vortexed briefly.

The denatured, reduced, blocked and digested peptides were labeled using 8-plex isobaric tagging for relative and absolute quantification (iTRAQ) [40]. To enable comparison of all 32 samples in 5 separate 8-plex analyses, 5/32 (19 µL) of each sample were pooled, split in 5 equal aliquots of 104 µL each, and were treated alongside the other samples to serve as internal standards of all 5 experimental sets (each standard labeled with iTRAQ-reagent 121).

After addition of 1 u iTRAQ-reagent per sample, tubes were incubated at 25 °C, under constant agitation, for 2 h. Following this, labeled samples of each 8-plex experimental set were pooled and centrifuged at 18.000 x g for 5 min. Supernatants were transferred to fresh tubes and pH was adjusted to 3.0-3.5 using HPLC-grade 5% trifluoroacetic acid (TFA). Tubes were incubated under constant agitation at 25 °C for 30 min before being dried in a speedvac overnight.

### Peptide separation and mass spectrometry

Labeled samples were prepared for mass spectrometric analysis as described previously [41]. Briefly, the 5 mixtures were subjected to two-dimensional liquid chromatography (LC). Multiple LC fractions of iTRAQ labeled peptides were captured, mixed with matrix and every two consecutive LC fractions deposited as 192 spots on a single MALDI plate. Mass spectrometry (MS/MS) was performed to identify peptides and quantify the iTRAQ signal using an ABI 4800 proteomics analyzer (Applied Biosystems). This procedure was repeated 5 times for all the biological independent tissue samples.

### Protein identification and statistical analyses

MS/MS spectra were searched against rat database using GPS Explorer (ABI) and Mascot (MatrixScience) with trypsin specificity and fixed iTRAQ modifications at lysine residues and N-termini of the peptides. Mass tolerance was 100 ppm for precursor ions and 0.5 Da for fragment ions; missed cleavage was allowed. For each MS/MS spectrum, a single peptide hit with the highest Mascot score in the Swissprot database (version 11/2011) was considered for further analysis. If a spectrum could not be annotated using Swissprot database, a second Mascot search was performed in the larger but more redundant NCBI database (version 11/28/2011). Next, the precursor protein sequences of all peptides from all 5 sets of samples were retrieved from the respective databases. NCBI sequences sharing more than 90% similarity over 85% of the sequence length with a Swissprot sequence were clustered as a single protein. Peptides that matched the sequence of multiple proteins were not removed from analyses. Proteins were included in analyses if at least 2 peptides had been identified with Mascot confidence > 95%, in addition to the criterion of at least 3 reliably quantified peptides (iTRAQ signature peak area above 500) in each of the 5 sets of samples. Individual peak areas of each iTRAQ signature peak of each peptide were log_2_-transformed, normalized to the average of all peak areas of the respective iTRAQ signature peak and mean centered. Within each set of samples, the abundance of a protein was calculated as the average of these mean centered iTRAQ values of multiple peptides, yielding 8 independent measurements of protein abundance for each of the 4 treatments.

Significance of treatment effects was evaluated using Student’s t-tests. To address the problem of multiple testing, resulting p-values were converted into q-values [42] giving an estimate of the false discovery rate (FDR) for each statistical test.

### Data analyses

Gene-Ontology (GO) – Analyses were performed using the online DAVID Bioinformatics Resources 6.7 tool [43,44] with a subset of rat hippocampal gene expression, provided by the Gene Expression Atlas of the European Bioinformatics institute [45,46], as background. P-values stated were calculated by the DAVID tool and were corrected according to Bonferroni.

### F-actin precipitation assay

The assay was adapted from Cenni et al., 2003 [47]. Briefly, human NLF neuroblastoma cells, grown to confluency and supplemented (30 min before lysis) with either DMSO only, 1 µM, or 5 µM of Mycalolide B (Santa Cruz Biotechnology, Inc., Heidelberg, Germany), an F-actin depolymerizing substance [48]. Cells were harvested from a 10 cm culture dish by trypsination and, subsequently, were washed twice with PBS. Then 0.4 mL of lysis- and F-actin stabilization buffer (50 mM Pipes, pH 6.9; 50 mM NaCl; 5 mM MgCl_2_; 5 mM EGTA; 5% [v/v] glycerol; 0.1% NP-40; 0.1% Triton X-100; 1% Tween 20; 1 x cOmplete, EDTA-free Protease Inhibitor Cocktail) was added to the pelleted cells and cells were lysed by pipetting repeatedly through a 200 µL pipette-tip. After incubating the lysate for 10 min at 37 °C, a 100 µL aliquot was centrifuged at 350 x g for 5 min to pellet any cell debris. The supernatant was then transferred into a 1.5 mL ultracentrifuge tube and was subjected to centrifugation at 100.000 x g for 60 min. The supernatant containing soluble proteins and unpolymerized G-actin was carefully removed and the pellet was re-solubilized in the same volume (100 µL). Both fractions were supplemented with SDS-loading buffer and were subjected to SDS-PAGE and subsequent immuno-blotting using an α-actin antibody (A2066; Sigma-Aldrich, Munich, Germany) and an α-ARP3 antibody (ab49671; Abcam, Cambridge, UK).

## Results

### Behavioral tests

Wild-type, adult and aged, male Wistar rats were characterized for their cognitive functions in the Morris-Water-Maze (MWM). For the cued version of the water maze, repeated measures ANOVA revealed a significant decrease in the distance covered to the cued platform over the four trials (F_3,84_ = 12.725, p ≤ 0.001), indicative of learning, but failed to reveal significant main effects for ‘age’ (F_1,28_ = 0.436, p = 0.515) or ‘learning performance’ (F_1,25_ = 0.018, p = 0.893; data not shown). These results indicate that sensory and motor deficits did not affect the groups differentially. Thus, group differences detected in the hidden platform task are rather attributable to differences in special learning capacities, than to sensory and motor deficits [35,49].

**Figure 1:**
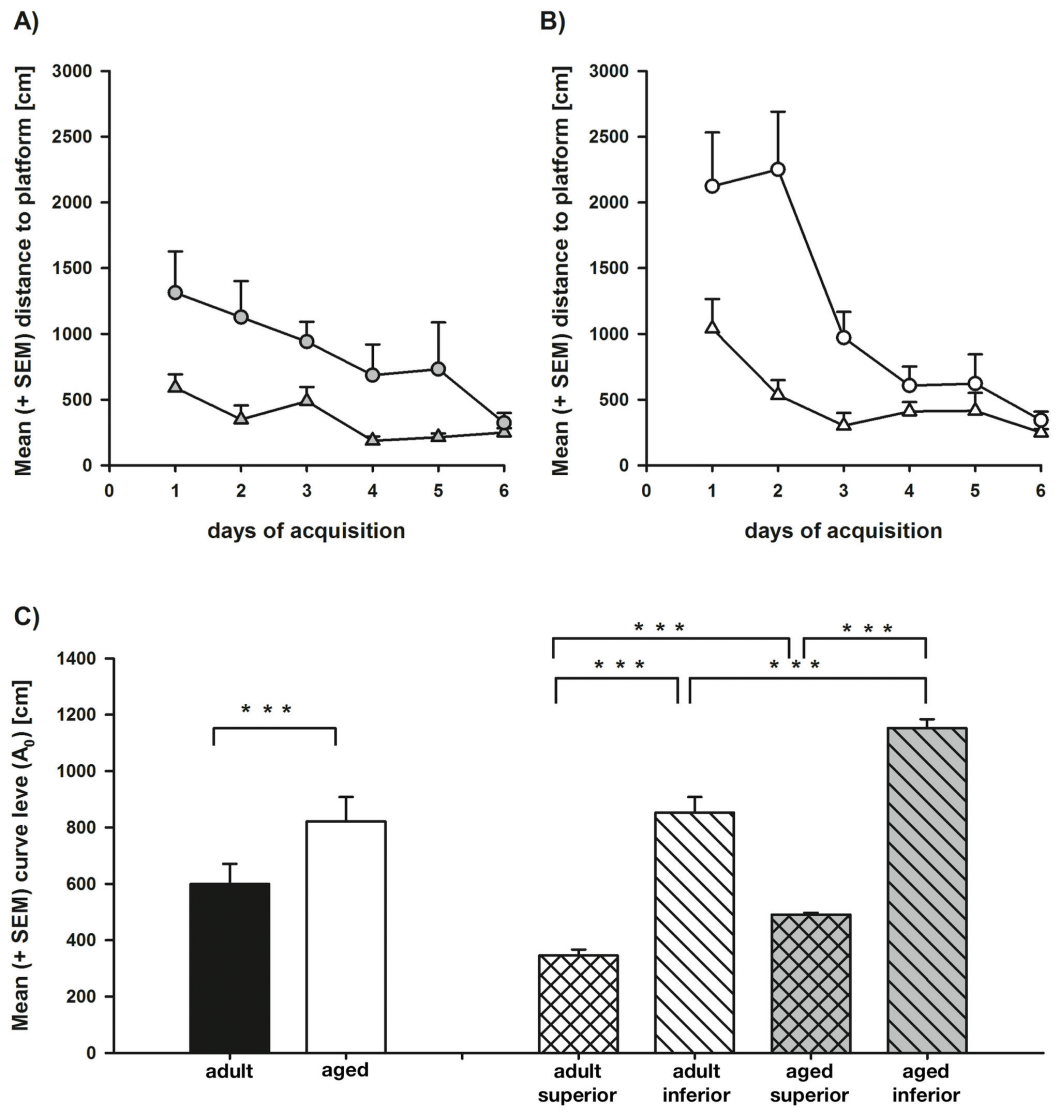
Cognitive performance as distance to platform during hidden platform place learning in the Morris water maze. Shown is the distance to the platform [cm] (+ SEM) for each acquisition day as averaged for adult superior (full triangles) and adult inferior (full circles) (**A**) as well as aged superior (open triangles) and aged inferior (open circles) (**B**) learners. **C**) Depicted is the mean curve level of the distance to platform (+ SEM) for the adult and aged rats as well as their subgroups adult superior, adult inferior, aged superior, and aged inferior learners (*, **, ***: p ≤ 0.05, 0.01, 0.001).

Results of the hidden platform task are summarized in Figure 1. Repeated measures ANOVA revealed a significant decrease in the mean distance covered to platform over the course of testing (F_5,140_ = 11.862, p ≤ 0.001), which significantly varied as a function of ‘learning performance’(F_5,140_ = 3.720, p = 0.003), but only marginally as a function of ‘age’ (F_5,140_ = 2.251, p = 0.053). Main effects were found for ‘age’ (F_1,28_ = 44.013, p ≤ 0.001) and ‘learning performance’ (F_1,28_ = 309.654, p ≤ 0.001). Also an interaction between ‘age’ and ‘learning performance’ became apparent (F_1,28_ = 5.727, p = 0.029).

Post-hoc analysis using t-tests showed that within each age group, the inferior performers, on average, moved longer distances to the hidden platform as compared to the superior performers (aged: t = -20.409; p ≤ 0.001; adult: t = -8.646; p ≤ 0.001). Furthermore, the aged inferior performers moved longer distances as compared to the adult inferior group (t = 4.725; p ≤ 0.001). Similarly, the aged superior group moved longer distances as compared to the adult superior group (t = -6.578; p ≤ 0.001). As to the slope (a_1_; data not depicted) over the hidden platform training trials, the aged inferior performers exhibited a stronger decrease in the distance to the hidden platform (and therefore a steeper slope a_1_) as compared to the aged superior group (t = 3.772; p = 0.002). However, such effects were not found when the adult inferior and the adult superior group were compared (t = 1.565; p = 0.140). Similarly, the aged superior performers did not differ from the adult superior performers in the slope a_1_ (t = -1.207; p = 0.247), but the aged inferior performers exhibited a steeper slope over the acquisition trials as compared to the respective adult performer group (t = -2.321; p = 0.036).

**Figure 2:**
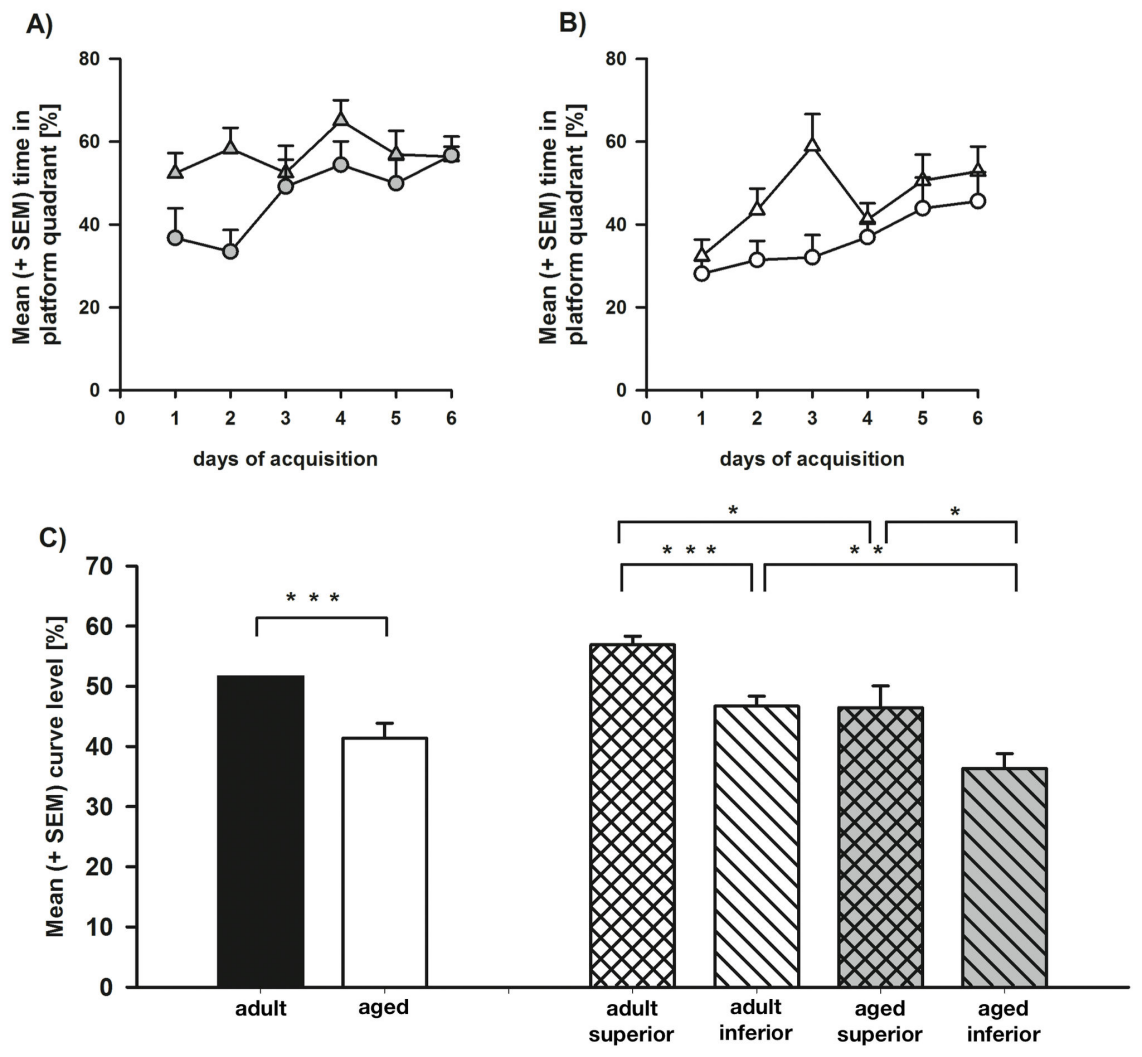
Cognitive performance place preference during hidden platform place learning in the Morris water maze. Shown is the time spent in the platform quadrant expressed as percentage of trial duration (+ SEM) for each acquisition day displayed by adult superior (full triangles) and adult inferior (full circles) (**A**) as well as aged superior (open triangles) and aged inferior (open circles) (**B**) learners. **C**) Depicted is the mean curve level [%] of the place preference (+ SEM) for adult and aged rats as well as adult superior, adult inferior, aged superior, and aged inferior learners (*, **, ***: p ≤ 0.05, 0.01, 0.001).

For the time spent in the platform quadrant (Figure 2), repeated measures ANOVA revealed a significant increase in the preference for the platform quadrant over the course of acquisition training (F_5,140_ = 4.688, p = 0.001), without any significant interaction effects (all F_5,140_ ≤ 1.612, all p ≥ 0.161). However, significant main effects were found for the factors ‘age’ (F_1,28_ = 18.131, p ≤ 0.001) and ‘learning performance’ (F_1,28_ = 17.315, p ≤ 0.001), indicating, that the groups also differed with respect to another direct measure of spatial learning, a preference for the reinforced platform quadrant. Post-hoc analysis using t-tests revealed that both superior performer groups exhibited a higher preference for the platform quadrant as compared to the respective inferior group of matched age (aged: t = 2.232, p = 0.036; adult: t = 4.649, p ≤ 0.001; Figure 2). Furthermore, adult inferior animals exhibited a stronger preference for the platform quadrant as compared to the aged inferior animals (t = -3.5, p = 0.004). Similarly, the adult superior rats showed a stronger preference for the platform quadrant as compared to the aged superior rats (t = -2.684, p = 0.018).

Repeated measures ANOVA revealed a significant decrease in speed of swimming over hidden platform place learning (F_5,140_ = 7.025, p ≤ 0.001), without any significant interaction effects (all F_5,140_ ≤ 2.046, all p ≥ 0.076). Furthermore, no significant main between-groups effects and interactions with the between-groups factors were revealed by ANOVA (all F_1,28_ ≤ 2.698, all p ≥ 0.112), ruling out the possibility that differences found during acquisition could be explained by differences in swimming speed.

### Proteomic analysis

**Figure 3:**
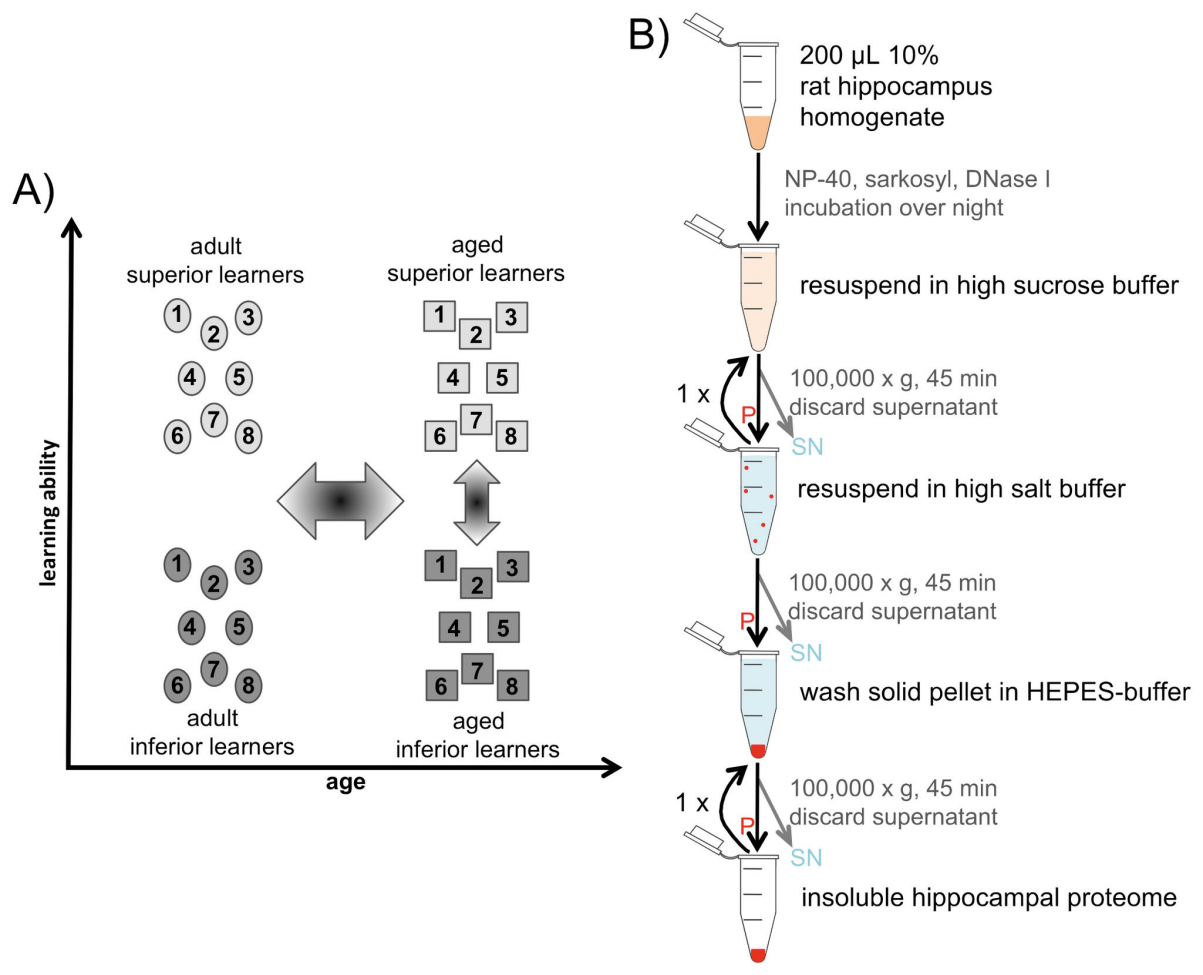
Grouping of animals and workflow. The left panel (**A**) displays the applied grouping of the rats according to their age and learning abilities, resulting in four groups of 8 individuals, each. The right panel (**B**) depicts the workflow of the insoluble proteome purification from rat hippocampal homogenate (red: insoluble protein components; P: pellet; SN: supernatant).

Brains from the 32 rats, each assigned to one of the four clusters ‘adult inferior’, ‘adult superior’, ‘aged inferior’, and ‘aged superior’ (Figure 3 A), were subjected to proteomic analysis. The hippocampus was selected as critical brain region since it is established to be essential for spatial memory [50] and is, therefore, an obvious brain region where molecular changes relating to impaired cognitive function, as assessed in MWM, could be present. For each of the four groups, hippocampal homogenates of the detergent-insoluble proteomes of 8 individual rats were purified to yield 32 aggregomes for quantitative mass-spectrometric analyses (Figure 3 B).

### Behavioral performance-specific hippocampal aggregome

**Figure 4:**
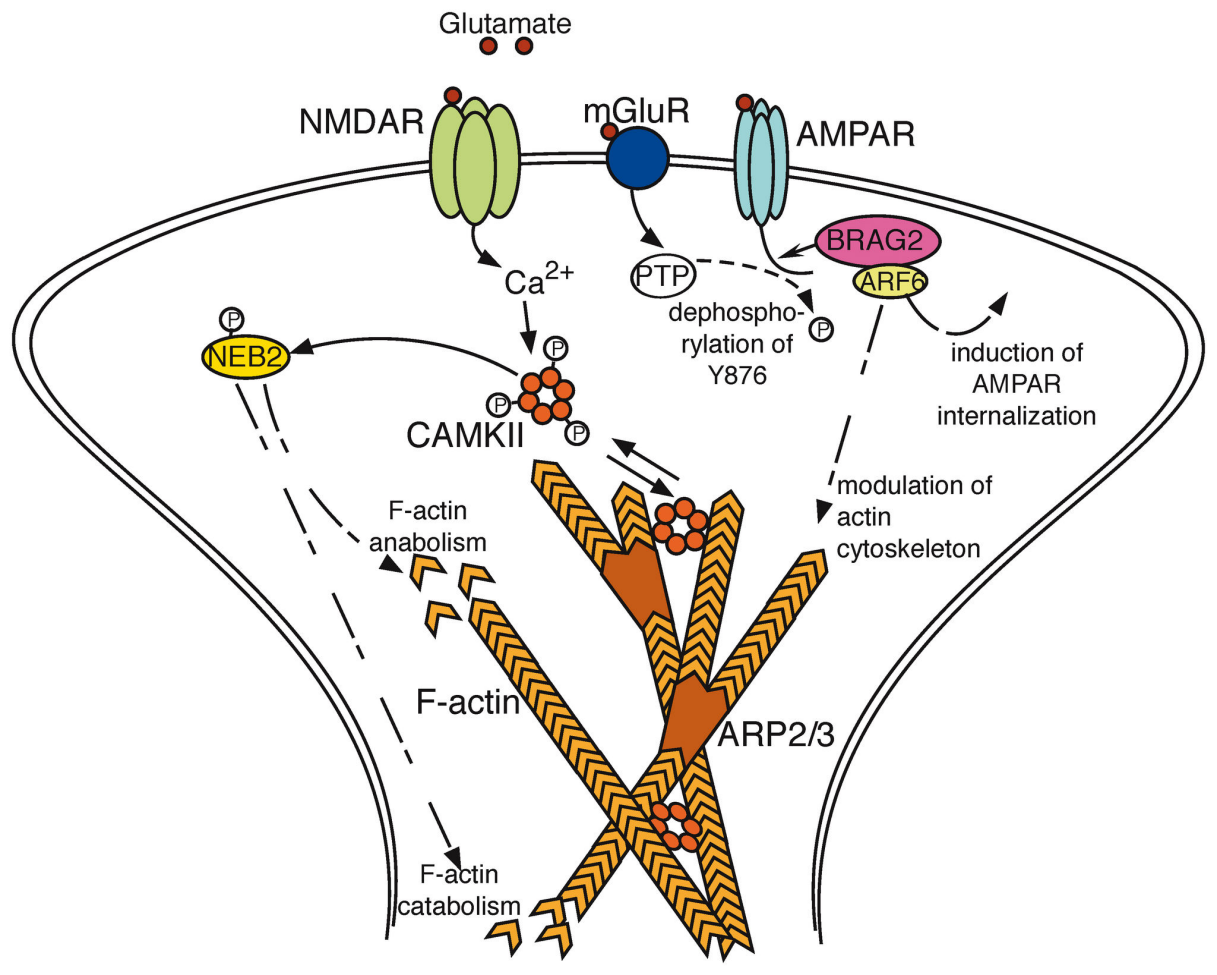
Structural plasticity in NMDA receptor-mediated long-term depression. Depicted is a scheme of NMDA receptor-induced LTD, bringing in context the presumably concerted actions of ARP3 – localized in the ARP2/3 complex; NEB2 – being activated via phosphorylation by CAMKII and acting on catabolic and anabolic processes on the filamentous actin network; and BRAG2 – binding to the AMPA receptor-GluA2 subunit upon its de-phosphorylation by the mGluR-activated protein tyrosine phosphatase (PTP) and inducing modulations of the actin cytoskeleton and internalization of AMPARs via its interaction with ARF6 [53,95]. Scheme based on Okamoto et al., 2009 [52], linking it to LTD [100] and including information concerning BRAG2 described by Scholz et al., 2010 [53] and summarized by Fitzjohn and Bashir, 2010 [95].

Only three proteins differed between aged superior and aged inferior learners in their segregation to the insoluble fraction (p < 0.05; Table 1). These proteins were identified as: Actin-related protein 3 (ARP3), spinophilin (neurabin-2, NEB2), and the IQ motif and SEC 7 domain-containing protein 1 (BRAG2). Most intriguingly, all three play crucial roles in synaptic plasticity, a phenomenon involved in memory formation [51,52,53] (Figure 4). We observed a decrease of insoluble ARP3 and NEB2, and an increase of insoluble BRAG2 in the aged inferior cohort, as compared to the group of aged superior animals.

**Table 1:** Top 10 proteins altered comparing the insoluble proteome of aged inferior and aged superior rats.

					**Pearson Correlation**			
		**aged inferior vs. aged superior**			**A_0_-distance (MWM**)		**a_1_-slope (MWM**)	
**Entry**	**Proteinname**	**Change**	**p-value**	**FDR**	**r**	**p**	**r**	**p**
ARP3_RAT	**Actin-related protein 3**	⬇	**0.0013**	0.2156	**-0.72**	**0.002**	**0.67**	**0.005**
NEB2_RAT	**Neurabin-2**	⬇	**0.0054**	0.4662	**-0.62**	**0.010**	0.47	0.066
gi|109473862	PREDICTED: IQ motif and Sec7 domain 1-like isoform 2 (**Brag2**)	⬆	**0.0310**	0.9328	**0.53**	**0.035**	**-0.73**	**0.001**
G3P_RAT	Glyceraldehyde-3-phosphate dehydrogenase	⬆	0.0547	0.9328	0.45	0.080	-0.55	0.027
MYH10_RAT	Myosin-10	⬆	0.0570	0.9328	-0.49	0.054	0.03	0.912
SHAN3_RAT	SH3 and multiple ankyrin repeat domains protein 3	⬇	0.0729	0.9328	-0.35	0.184	0.31	0.243
ACTB_RAT	Actin, cytoplasmic 1	⬇	0.0841	0.9328	-0.43	0.096	0.38	0.147
DYHC1_RAT	Cytoplasmic dynein 1 heavy chain 1	⬆	0.0861	0.9328	0.40	0.125	-0.33	0.212
AGAP2_RAT	Arf-GAP, GTPase, ANK repeat and PH domain-containing protein	⬆	0.0965	0.9328	0.46	0.073	-0.47	0.066
SYGP1_RAT	Ras GTPase-activating protein SynGAP	⬆	0.1008	0.9328	0.43	0.096	-0.58	0.019

Values in bold are significant. Arrow down: Lower abundance in aged inferior. Arrow up: Higher abundance in aged inferior. p: p-value. r: Pearson’s correlation coefficient. MWM: Morris Water Maze. A_0_-distance: Total distance swam to hidden platform. a_1_-slope: Slope of the A_0_ performance curve; learning pace.

**Figure 5:**
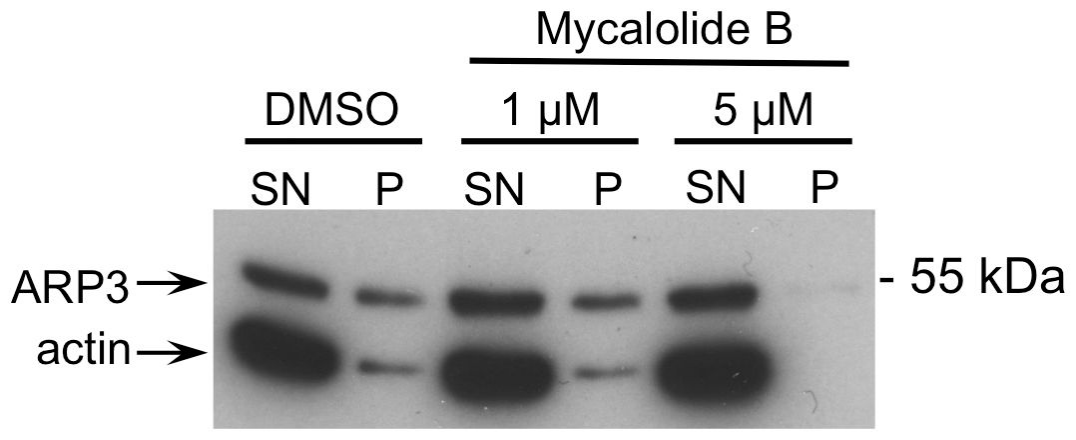
F-actin precipitation assay. Western blot of the supernatant (SN) and insoluble pellet (P) fraction of cell lysates subjected to an F-actin precipitation assay. Visible are immunostained bands of actin and ARP3 as designated. Cells were pre-treated with 1 µM or 5 µM of the F-actin de-polymerizing agent Mycalolide B, or with the respective amount of the solvent DMSO only. Upon treatment with 5 µM Mycalolide B, actin (F-actin) and ARP3 simultaneously disappear from the pellet fraction indicating that ARP3’s insolubility is dependent on F-actin.

To investigate a possible mechanism by which a higher level of a specific protein in the insoluble fraction of superior performers could be explained, we tested whether insolubility could be mediated by other proteins, more prone to sedimentation. Inspired by the joint role of the identified proteins ARP3, NEB2, and BRAG2 in synaptic plasticity, where polymerization of actin monomers to F-actin polymers plays a critical role, we performed F-actin precipitation assays and were able to validate a co-precipitation of ARP3 with F-actin (Figure 5). We could demonstrate ARP3 co-precipitation at a centrifugation speed of 100.000 x g with an intact F-actin network (Figure 5, DMSO control) but not after F-actin depolymerization by Mycalolide B (Figure 5, 5 µM Mycalolide B).

**Figure 6:**
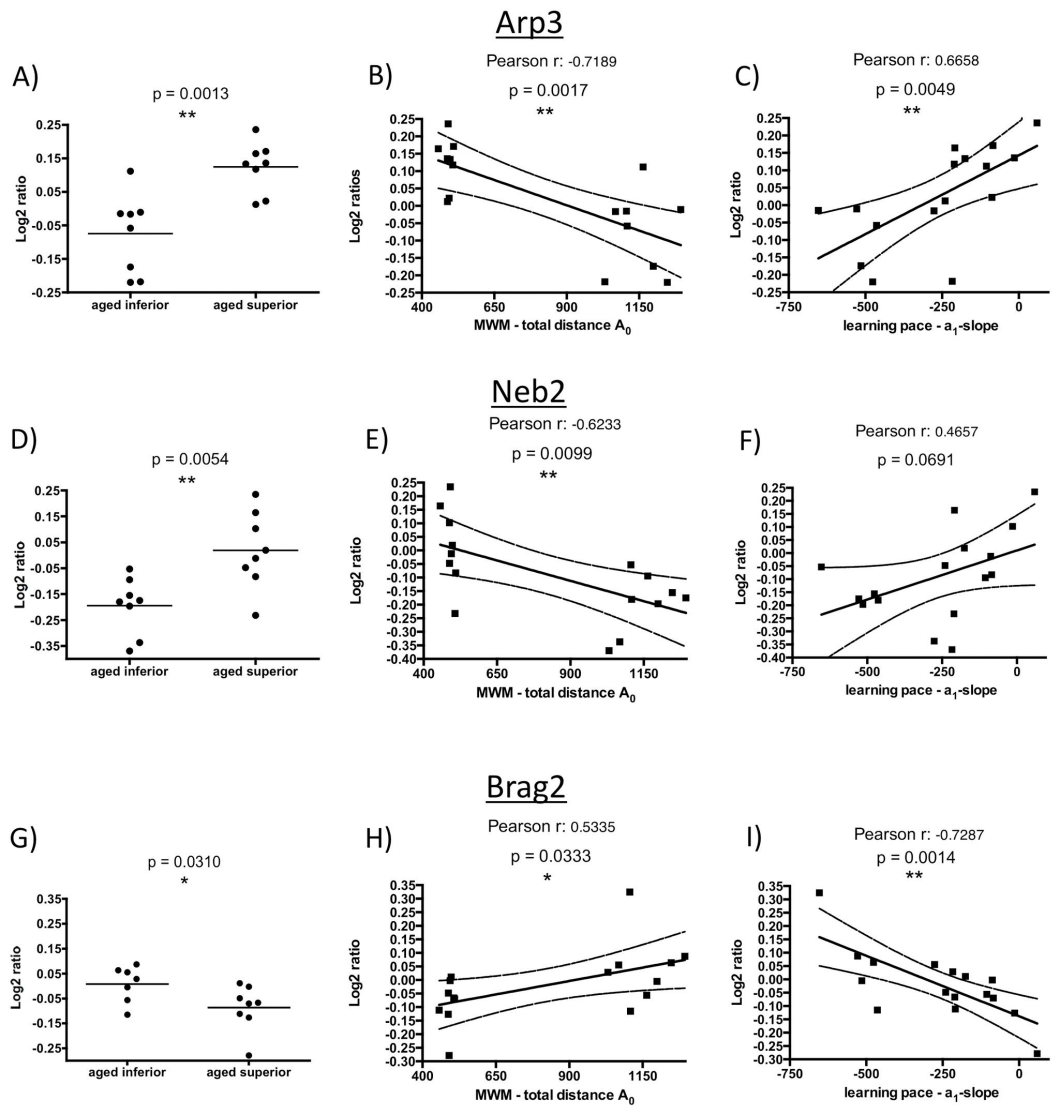
Relative abundance of insoluble ARP3, NEB2, and BRAG2 comparing aged superior and aged inferior learners. Displayed are the log_2_-transformed relative abundances of each of the three proteins as determined for all 16 aged rats by iTRAQ mass spectrometry. Graphs **A**, **D**, and **G** show a group-wise comparison of aged superior and the aged inferior rats. Panels **B**, **E**, and **H** display the log_2_ protein values plotted against the rats’ performance in the MWM assessed as mean total distance A_0_ [cm]. Drawn in graphs C, F, and **I** are the log_2_ protein values plotted against the rats’ performance in the MWM as the learning pace, assessed as a_1_-slope over the 6 trial days. Besides the Pearson correlation values (r) and the respective p-value, graphs B, C, **E**, F, H, and **I** also display the line of linear regression along with its 95%-confidence interval.

In addition to the detected changes in levels of ARP3, NEB2, and BRAG2, we also found correlations between these 3 proteins and the respective cognitive performances of the individual aged rats, as assessed by total mean swimming distance (A_0_) to the hidden platform in the MWM (Table 1, Figure 6 B, E, H) corroborating their critical role in maintaining cognitive performance. ARP3 and NEB2 accumulation correlated negatively with A_0_, contrary to the concentration of insoluble BRAG2. Furthermore, ARP3 and BRAG2 showed correlations to the learning rate a_1_ of the individual aged animals (Table 1, Figure 6 C, F, I), with ARP3 displaying a positive correlation and BRAG2 being negatively correlated with a_1_. For NEB2, the calculated (positive) correlation exceeded the significance threshold (p = 0.066).

**Figure 7:**
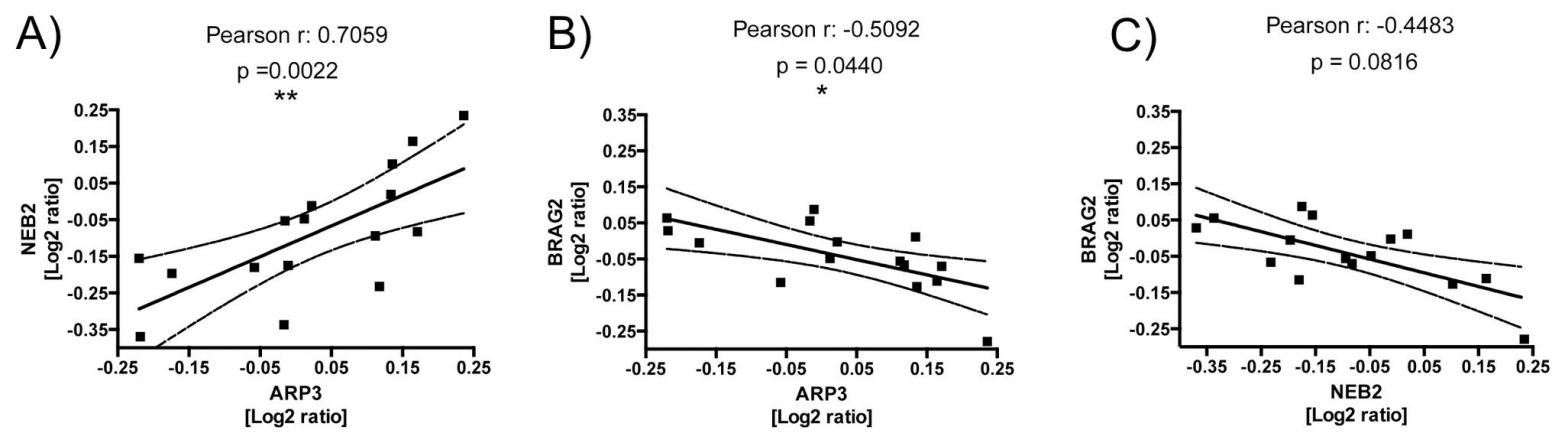
Protein inter-correlations. Displayed are the log_2_-transformed relative abundances of ARP3, NEB2, and BRAG2 plotted against one another to visualize possible correlative changes. Graphs show NEB2-values plotted against ARP3-values (**A**), BRAG2 against ARP3 (**B**), and BRAG2 against NEB2 (**C**). Besides the Pearson correlation values (r) and the respective p-value, graphs also display the line of linear regression along with its 95%-confidence interval.

The amount of ARP3 protein in the detergent-insoluble fractions of aged rats also correlated positively with the amount of NEB2 protein (Figure 7 A) identified in the same fractions. ARP3 showed a negative correlation with BRAG2 in the aged cohort (Figure 7 B), consistent with the inverse accumulation of BRAG2 and its diverse role in the post-synapse. A similar correlation was found for all aged and adult rats combined (r = -0.377, p = 0.034; data not shown).

### Age-specific hippocampal aggregome

Comparing the aged and adult rat cohort, we identified 52 insoluble proteins that differed as a function of age (p < 0.05, FDR < 11.7%; Table 2). Amongst those proteins enriched significantly in the insoluble fraction of the aged (p < 0.014; FDR < 6.7%), the cluster of microtubule-associated proteins (dynactin subunit 1, cytoplasmic dynein 1 heavy chain 1, microtubule-associated protein 2, tubulin alpha-4A chain) was represented most prominently in aged compared to adult rats with a 20-fold enrichment (p = 0.035), as determined by gene ontology (GO) clustering analyses. Decreases in the insoluble fraction (p ≤ 0.014) on the other hand, were found for members of the post-synaptic density, PSD, (disks large homolog 2, glutamate [NMDA] receptor subunit epsilon-1, SH3 and multiple ankyrin repeat domains protein 1-3), that were enriched by 45-fold (p = 9.7 x 10^-5^). The additional identified GO-cluster of general ‘cytoskeleton’ (especially actin) associated proteins, extended the PSD-cluster by the proteins actin-related protein 2/3 complex subunit 2, protein bassoon and SRC kinase signaling inhibitor 1. These proteins of the ‘cytoskeleton’ GO-cluster were found 8-fold enriched among the set of decreased proteins (p = 2.1 x 10^-4^).

**Table 2:** Proteins significantly altered comparing the insoluble proteome of aged and adult rat hippocampi.

		**aged vs. adult**		
**Entry**	**Proteinname**	**Change**	**p-value**	**FDR**
HPLN2_RAT	Hyaluronan and proteoglycan link protein 2	⬆	2.6 x 10^-9^	2.3 x 10^-7^
GFAP_RAT	Glial fibrillary acidic protein	⬆	2.0 x 10^-6^	0.00012
MOBP_RAT	Myelin-associated oligodendrocyte basic protein	⬆	5.5 x 10^-6^	0.00023
DCTN1_RAT	Dynactin subunit 1	⬆	2.8 x 10^-5^	0.00089
BSN_RAT	Protein bassoon	⬇	4.9 x 10^-5^	0.00126
SHAN3_RAT	SH3 and multiple ankyrin repeat domains protein 3	⬇	8.6 x 10^-5^	0.00184
HOME1_RAT	Homer protein homolog 1	⬇	0.00022	0.00406
SNIP_RAT	SRC kinase signaling inhibitor 1	⬇	0.00026	0.00414
AGAP2_RAT	Arf-GAP, GTPase, ANK repeat and PH domain-containing protein 2	⬇	0.00062	0.00881
gi|293342552	PREDICTED: collagen, type IV, alpha 2	⬆	0.00111	0.01414
SHAN1_RAT	SH3 and multiple ankyrin repeat domains protein 1	⬇	0.00122	0.01418
ERC2_RAT	ERC protein 2	⬇	0.00169	0.01760
CN37_RAT	2', 3'-cyclic-nucleotide 3'-phosphodiesterase	⬆	0.00179	0.01760
TBA4A_RAT	Tubulin alpha-4A chain	⬆	0.00247	0.02255
GLNA_RAT	Glutamine synthetase	⬆	0.00326	0.02778
DLG2_RAT	Disks large homolog 2	⬇	0.00377	0.03014
G3P_RAT	Glyceraldehyde-3-phosphate dehydrogenase	⬆	0.00455	0.03426
BAIP2_RAT	Brain-specific angiogenesis inhibitor 1-associated protein 2	⬇	0.00675	0.04665
SHAN2_RAT	SH3 and multiple ankyrin repeat domains protein 2	⬇	0.00693	0.04665
DYHC1_RAT	Cytoplasmic dynein 1 heavy chain 1	⬆	0.00812	0.04889
gi|157818467	Heat shock 70kDa protein 12A	⬆	0.00813	0.04889
gi|293345780	PREDICTED: similar to Gene model 996	⬇	0.00841	0.04889
SYN1_RAT	Synapsin-1	⬇	0.01125	0.06007
MTAP2_RAT	Microtubule-associated protein 2	⬆	0.01127	0.06007
IDH3B_RAT	Isocitrate dehydrogenase [NAD] subunit beta, mitochondrial	⬆	0.01396	0.06635
NMDE1_RAT	Glutamate [NMDA] receptor subunit epsilon-1	⬇	0.01397	0.06635
ARPC2_RAT	Actin-related protein 2/3 complex subunit 2	⬇	0.01400	0.06635
DLGP2_RAT	Disks large-associated protein 2	⬇	0.01614	0.07203
DPYL2_RAT	Dihydropyrimidinase-related protein 2	⬇	0.01707	0.07203
gi|157823479	Prickle homolog 2	⬇	0.01731	0.07203
gi|33563266	NADH dehydrogenase (ubiquinone) 1 alpha subcomplex, 4	⬆	0.01745	0.07203
DLGP3_RAT	Disks large-associated protein 3	⬇	0.01835	0.07289
NEB2_RAT	Neurabin-2	⬇	0.01937	0.07289
NCAN_RAT	Neurocan core protein	⬆	0.01992	0.07289
EF2_RAT	Elongation factor 2	⬆	0.01994	0.07289
ATPA_RAT	ATP synthase subunit alpha, mitochondrial	⬆	0.02084	0.07365
HPLN1_RAT	Hyaluronan and proteoglycan link protein 1	⬆	0.02130	0.07365
MYO1D_RAT	Myosin-Id	⬆	0.02308	0.07773
GELS_RAT	Gelsolin	⬆	0.02684	0.08348
TBB2C_RAT	Tubulin beta-2C chain	⬆	0.02721	0.08348
RL15_RAT	60S ribosomal protein L15	⬇	0.02740	0.08348
DYN1_RAT	Dynamin-1	⬇	0.02544	0.08348
gi|5031595	Actin related protein 2/3 complex, subunit 4 [Mus musculus]	⬇	0.02912	0.08667
AT1A1_RAT	Sodium/potassium-transporting ATPase subunit alpha-1	⬆	0.03096	0.09005
RL7A_RAT	60S ribosomal protein L7a	⬇	0.03515	0.09773
NMDE2_RAT	Glutamate [NMDA] receptor subunit epsilon-2	⬇	0.03535	0.09773
BEGIN_RAT	Brain-enriched guanylate kinase-associated protein	⬇	0.03589	0.09773
NSF_RAT	Vesicle-fusing ATPase	⬆	0.04010	0.10547
gi|149066065	rCG59984	⬇	0.04039	0.10547
TBB3_RAT	Tubulin beta-3 chain	⬆	0.04307	0.11022
DLG4_RAT	Disks large homolog 4	⬇	0.04726	0.11634
KCC2A_RAT	Calcium/calmodulin-dependent protein kinase type II alpha chain	⬇	0.04728	0.11634

Arrow down: Lower abundance in the aged. Arrow up: Higher abundance in the aged. FDR: False discovery rate.

## Discussion and Conclusions

In this study, we demonstrate in a quantitative proteomics (iTRAQ) approach that the analysis of changes in the insoluble proteome of the hippocampus identifies molecular components correlating with functional systems data, here the animal’s age and cognitive performance in a learning and memory task (MWM).

Our study is the first proteome-wide approach describing insoluble protein composition in the aged mammalian brain and relates to previous studies performed in the nematode *Caenorhabditis elegans* where changes in protein solubility in the course of aging were detected [19,20]. In those studies, detergent-insoluble proteins were purified from young and old, pooled whole *C. elegans*, and protein components of the aggregomes were quantified using iTRAQ. Compared to the studies of David et al. and Reis-Rodrigues et al. in *C. elegans* where changes above 10-fold were reported [19,20], the range of the detected differences between groups in our study was much smaller. One explanation for this may be the presence of different protein degradation mechanisms in mammalian cells as compared to nematodes, allowing for a more efficient clearing of protein aggregates [54], or the specific focus on a brain subregion in our study, as opposed to using the whole organism. In addition, there are important technical differences between the *C. elegans* studies and our investigations. In our study on the mammalian brain, we chose an approach with high statistical power (8 vs. 8 and 16 vs. 16 biological replicates for ‘cognition in aged’ and ‘aging’, respectively) compared to smaller numbers of biological replicates (n = 2-4) used in the investigations on whole *C. elegans* [19,20]. We noted that, despite normalization to input tissue-weight before purification, similar to previous studies, strong variances in total detected protein levels were detected between single animals after purification, exceeding the differences in protein levels between the analyzed groups. Therefore, for the present investigation, we had to adopt a global normalization method prior to statistical testing. Finally, the aggregome purification protocols applied in the *C. elegans* studies were distinct from ours and changes in the use of detergents, centrifugation speed, and a final extraction with formic acid, may result in differences in the set of proteins identified in the aggregome. Compared to David et al. [20], we used higher centrifugation speed (100.000 x g instead of 20.000 x g), applied less ionic detergent (0.2% sarkosyl instead of 1% SDS/SDO), and performed two subsequent lipid and low molecular weight protein extractions in high-density buffer (1.1 and 1.6 M sucrose instead of 1.0 M). Furthermore, we used 1.5 M NaCl instead of 0.75 M in our high ionic strength buffer, and did not apply a final extraction in 70% formic acid, that was introduced by David and co-workers to exclude their nematode cuticular debris from the analyses. Reis-Rodriguez and colleagues [19] pre-cleared their samples by centrifugation at 3.000 x g and reduced the purification protocol to three subsequent washes with 1% SDS and centrifugation steps at 16.000 x g, followed by a final extraction with 70% formic acid.

For our work, in order to include more potentially altered proteins in our post-hoc analyses, we decided, contingent upon statistical significance, to be less restrictive with regard to the FDRs (listed in Tables 1 and 2). For the proteins associated with ‘aging’, this resulted in the inclusion of proteins showing an FDR of 0.1163, whilst for the ‘cognition in aged’ analyses all three proteins identified as changed with p ≤ 0.05 were analyzed further.

### Proteins identified in the insoluble fraction of superior vs. inferior performing aged rats

ARP3 and NEB2 proteins were identified at higher concentrations in the insoluble fraction of the aged superior performers as compared to their age-matched impaired, inferior performers. In the context of synaptic plasticity, a likely explanation is that with our biochemical fractionation we pull down significant amounts of dendritic F-actin (see also the likely increased pull-down of insoluble actin in superior performers; Table 1) and associated proteins like ARP3 (Figure 5). However, other reasons such as specific post-translational modifications, or secondary effects like a specific local increase in protein density with a change in facultative protein-protein interactions should also be considered. In contrast, BRAG2, being critical for the induction of LTD [53], accumulated in the aggregome of the aged inferior learners. The observed inverse correlation of BRAG2 with ARP3 (Figure 7 B) seems conclusive with its rather inverse role in synaptic plasticity.

The solubility changes of ARP3, NEB2, and BRAG2 detected in our proteomic analysis of aged rats are converging on an inter-related set of proteins involved in synaptic plasticity. This becomes even more intriguing, as these changes not only showed significance in the grouped comparison of aged superior and inferior performers, but, additionally, proved correlative for the cognitive performances (A_0_) of the individual animals (Figure 6 B, E, H), as well as – in the case of ARP3 and BRAG2 – for the individual learning rate a_1_ (Figure 6 C, I). Also inter-correlation of the solubility changes between NEB2 and ARP3, as well as between ARP3 and BRAG2 were observed (Figure 7). For ARP3, coprecipitation with immobilized NEB2 has been described [55].

So far, these proteins have not been reported in the context of changes in cognitive performance in analyses of whole synaptic protein fractions, i.e. comprising soluble and insoluble conformers [14,15,17,56,57,58]. Notably, in one study investigating differences in the whole hippocampal proteome of mice trained in different spatial memory tasks, Zheng and co-workers (2009) [16] found that ARP3, along with ARPC5 and F-actin-capping protein subunit beta, was differentially regulated in mice trained in different tasks. However, in their approach, changes were only observed comparing the different means of training, but did not correlate with any learning behavior within the trials.

The term synaptic plasticity describes structural and functional changes of dendritic spines and their postsynaptic densities (PSDs). These changes are observed following learning or the experimental induction of long-term potentiation (LTP) and long-term depression (LTD), respectively, and are believed to contribute to memory formation (reviewed by Lamprecht and LeDoux, 2004 [59]). LTP has been directly linked to performance in learning tasks such as the MWM [30], and thus, can be seen as a cellular manifestation of the observed rat behavior in learning and memory [60]. Basis of LTP and LTD are changes in the molecular composition of key proteins (e.g. AMPA receptors [61,62]) and cellular structures at synapses [52]. Both, LTP and LTD lead to the re-modeling of the actin cytoskeleton in dendritic spines [52,63]. Whereas LTP initializes the introduction of receptors into the postsynaptic membrane [64], its counter-player process LTD, induced by NMDA receptor (NMDAR) and metabotropic glutamate receptor (mGluR) activity [65], has been shown to evoke internalization of AMPA receptors (AMPARs) [64]. Like LTP, LTD is generally believed to play a crucial role in hippocampal memory formation [66].

The modulation of dendritic spine volume to mediate synaptic structural plasticity mainly involves the reorganization of the spine’s actin-cytoskeleton, which is mediated by signaling proteins such as Neurabin-2 (Spinophilin) and the Ca^2+^/calmodulin-dependent protein kinase, CAMKII [52] (Figure 4). This remodeling and the extension or degradation of the F-actin (filamentous actin) network in dendritic spines is crucial for synaptic plasticity and LTP/LTD maintenance [51,67,68], and there is a direct and crucial link of this to learning and memory formation [51,59,67,68,69,70,71].

#### ARP3

Actin-related protein 3 (ARP3) was found elevated in the insoluble fraction of hippocampi from aged rats displaying superior cognitive abilities. ARP3 expression is crucial to embryonic viability past the blastocyst stage [72] and is part of a protein complex, which includes ARP2 and the five subunits ARPC1-5 [73,74,75,76,77]. The complex builds the branching points of F-actin filaments [76,78] and thereby mediates the formation of branched structures within the actin cytoskeleton network [76,79] (Figure 4). Showing high concentrations in dendritic spines [80], the ARP2/3 complex is responsible for the actin network organization in spine heads and disturbances in expression of its subunits results in impaired spine and synapse formation [81,82], and in changes of synapse activity [81].

#### NEB2

The second protein identified as accumulating in the ‘aged superior’ aggregome, is spinophilin, also termed neurabin 2 (NEB2). It is a protein phosphatase I (PP-I) interacting and PP-I – activity modulating protein [83,84] that is primarily found in dendritic spines [83,85]. Intraspinal localization of NEB2 and its F-actin binding and bundling capacity were demonstrated to be modulated via its phosphorylation by the Ca^2+^/calmodulin dependent kinase II (CAMKII) [86] or the protein kinase A (PKA) [87], linking spinophilin action with its responsiveness to NMDA and adrenergic receptor activity [88,89] (Figure 4). The NEB2-mediated effect on F-actin organization within dendritic spines, thus, depends on Ca^2+^ as well as on cAMP signaling. It is, presumably, via modulation of the spinal F-actin network, that NEB2 modulates dendritic morphology [90] and, hence, has been found to be important for hippocampal integrity [90]. Notably, despite its effect on F-actin organization, mice, deficient in spinophilin, showed no altered LTP, but reduced LTD [90].

Previous experiments investigating a direct quantitative relation between NEB2, aging and cognitive abilities showed no positive results for the total and unfractionated protein levels [91,92]. These results, compared to our findings reported here, highlight the necessity to differentiate the solubility status of synaptic proteins for determining their function.

#### BRAG2

The third protein identified in our study comparing aged inferior and superior rats, BRAG2, was found increased in the aggregome of aged inferior rats. First described by Someya et al. in 2001 [93], this protein features an IQ-like motive and a SEC7 domain, and acts as a guanine nucleotide-exchange protein for the ADP-ribosylation factor 6 (ARF6) and, hence, is also termed IQSEC1 or ARF-GEP100. Like the other two proteins found to be changed with cognitive ability in the aggregome of aged rats – ARP3 and NEB2 – BRAG2 has been implicated in the mechanism of actin-remodeling [94] and a direct effect on LTD-maintenance has been observed [53]. NMDAR- as well as mGluR-mediated LTD was found to rely upon BRAG2 expression [53] and a mechanism of BRAG2 binding to the GluA2 C-terminal part of AMPA receptors to induce their ARF6 mediated internalization [53,95] along with changes in the actin cytoskeleton [94], has been described (Figure 4).

In our proteomic results on BRAG2, one value (at 0.325) for the aged inferior cohort appeared to be standing out amongst the other values. Hence, a critical outlier-consideration was performed, where the value passed the ROUT and Grubb’s test for outliers with stringency set to 5%. Furthermore, no other data derived from this particular rat appeared to be irregular. Therefore, the value was included in the post-hoc analyses. Yet, even if excluded from the analysis, the comparison of aged inferior and aged superior rats in their amounts of insoluble BRAG2 still passed the significance threshold with p = 0.0449 (data not shown).

### Proteins identified in the insoluble fraction of aged vs. adult rats

In the approach by David et al., an overall number of 461 insoluble proteins was found increased by more than two-fold in the aged samples [20], whereas Reis-Rodrigues et al. reported the finding of 203 insoluble proteins [19]. Elongation factor 2, heat shock protein 70, and glyceraldehyde-3-phosphate dehydrogenase showed high abundance for the aged samples both in the insoluble fractions for the nematode approaches [19,20] and in our rat hippocampal fraction. In addition, the work of David and co-workers shared the detection of up-regulated myosins with the present study [20]. The study conducted by Reis-Rodrigues et al. reported findings – similar to our results for rat hippocampi – of elevated levels of aggregated tubulin and mitochondrial ATP synthase subunit alpha in aged worms [19], with the latter protein component having been recognized as a common denominator in the aging-pigment lipofuscin [96,97]. The increase of 60S ribosomal protein in the insoluble fraction of the aged animals, observed in the nematode studies [19,20], however, opposes our findings of a decrease in aged rat brain aggregome.

Reis-Rodrigues and colleagues further reported the finding of significant extensions of lifespan of *C. elegans* upon RNAi-based knockdown for almost half of the genes tested and whose products were found to be elevated in the aged aggregome [19]. Amongst those were the elongation factor 2, implicated in the ribosomal translation elongation, and tubulin, a component of the microtubular cytoskeleton [19].

Notably, there is no large overlap in the proteome from the insoluble fraction used in this study, compared to the aged rat hippocampal synaptoproteome described by Van Guilder and colleagues [12]. This corroborates the expected specificity of proteins prone to aggregate with age and accounts for the solubility changes to not be mere mirroring effects of altered expression levels, but rather to reflect changes in the proteostasis maintaining cellular machinery. However, some commonalities could be described: Whereas dynamin-1, which appears to be downregulated in the aged rat synaptic proteome [12] and was also less abundant in the aged aggregome, other proteins rather showed an opposite trend. Synapsin-1, a phosphoprotein associated with synaptic vesicles [98], was reported to show elevated expression comparing aged and adult synaptoproteome [12], but was reduced in the aged aggregome. Heat shock protein 70, isocitrate dehydrogenase [NAD], NADH dehydrogenase (ubiquinone), and tubulins have been demonstrated to be less abundant in the soluble aged rat hippocampus synaptoproteome [12], whereas they showed elevated quantities in the detergent-insoluble fraction analyzed in this present study. This latter, inverse correlation may point towards proteins, that specifically become less soluble with age, e.g. by oxidative stress [99], and are therefore depleted of the soluble fraction analyzed by Van Guilder and colleagues [12].

In this study, we demonstrated age-associated changes of protein solubility in the mammalian hippocampus of the rat. We used quantitative proteomics to also differentiate inferior and superior performing aged rats in a functional assay of memory and identified three proteins ARP3, NEB2, and BRAG2 involved in synaptic plasticity and LTD as potential molecular correlates of the age-associated memory decline. We thereby demonstrated that quantitative proteomics of the aggregome is an appropriate method for identifying molecular components of behavior associated with memory/learning processes in a systems biology approach to studying the aged brain.
