# Correction: Aging-Induced Proteostatic Changes in the Rat Hippocampus Identify ARP3, NEB2 and BRAG2 as a Molecular Circuitry for Cognitive Impairment

**DOI:** 10.1371/annotation/2546f4c2-b07f-4450-8d3c-f3ee81127b4a

**Published:** 2013-10-09

**Authors:** Philipp Ottis, Bianca Topic, Maarten Loos, Ka Wan Li, Maria A. de Souza Silva, Daniela Schulz, August B. Smit, Joseph P. Huston, Carsten Korth

The name of the fifth author was given incorrectly. The correct name is: Maria A. de Souza Silva. 

The correct citation is: Ottis P, Topic B, Loos M, Li KW, de Souza Silva MA, et al. (2013) Aging-Induced Proteostatic Changes in the Rat Hippocampus Identify ARP3, NEB2 and BRAG2 as a Molecular Circuitry for Cognitive Impairment. PLoS ONE 8(9): e75112. doi:10.1371/journal.pone.0075112. 

The correct abbreviation in the Author Contributions statement is: MAdSS. 

